# Effects of Tobacco

**Published:** 1873-02

**Authors:** 


### ARTICLE IX.

### *Effects of Tobacco.*

The subject of the effects of tobacco smoking has again become the topic of controversy, and several papers have recently been published upon it. Our readers will probably remember the discussion which was some years carried on in the daily papers in regard to its advantages, and which was terminated by an excellent letter to *The Times* from Sir Benjamin Brodie, in which he pointed out that, whilst it was usually a bad habit only, it might under circum stances of privation or exhaustion prove of extreme value in soothing and tranquilizing the nervous system. Dr. Hirt, in his admirable "*Krankheiten der Arbeiter*," or "Diseases of Artisans," tells us that the older writers on the action of tobacco manufacture—as Ramazzini, Cadet, Gassicourt, Fourcroy, Merat, and Tourtel—regarded the "weed" as highly pernicious, shortening life, and occasioning all kinds of diseases. The careful observation of Thackrah, however, corroborated by those of Parent-Duchatelet and D'Arcet, dispelled these gloomy views by showing that, as a rule, the workers in tobacco are remarkably free from pulmonary disease (except during their first exposure to the dust, when they suffer from bronchial catarrh,) and, in fact, are exceptionally healthy.

However this may be in regard to those engaged in the manufacture of tobacco, it is certain that excess in smoking exercises a very deleterious influence upon the economy at large, and especially upon the nervous system, as is clearly evidenced by the palid complexion and trembling hands of the confirmed smoker, to say nothing of the peculiar form of amaurosis which is generally admitted by opthalmic surgeons to result from it. What the precise agent may be by which these effects are produced appears to be at present somewhat doubtful. Up to a very recent period it was all but universally allowed to be due to the action of the nicotine contained in the leaf, which was considered to be volatilisable, and to be inhaled with the smoke; but lately some good chemists—as Zeise, Melsens, and Vohl—have been unable to find more than a trace of nicotine in the smoke, and have suggested that the injurious effects of tobacco are in reality due to the picolin bases, which are the final results of the decomposition of nicotine by slow combustion.

An essay that has just been published in the *Centralblatt* by Dr. Heubel, of Kiew, seems to prove the correctness of the older view; for he found that, by collecting the smoke of a few cigars in a Liebig's refrigerator, or by transmitting it through distilled water or alcohol, he could obtain a brownish liquid of pungent taste, alkaline reaction, and the well known odor of tobacco, which possessed all the characteristic toxic actions of nicotine. Frogs into which a few drops, of this fluid were subcutaneously injected gave unmistakable signs of tobacco poisoning, in their peculiar convulsions, and in the gradual progressive paralysis of their nerve-centres and motor nerves. In guinea pigs and rabbits the injection of a few drops caused great contraction of the pupils, which was confirmatory evidence of the presence of nicotine.

Heubel's experiments further demonstrate the important fact that, although nicotine is itself highly volatile, its salts —as the tartrate, acetate, sulphate, &c.—preserve their characteristic poisonous qualities when vaporated to dryness in the water bath. It appears to exist in the leaf in the forms of malate and citrate. These experiments, however, do not settle the question as to whether there may not be other constituents in tobacco smoke that are capable of acting prejudicially upon the body. Vogel and Reischauer some time ago showed the presence of sulphide and cyanide of ammonia, and it is quite possible that some of the peculiar effects of tobacco may be due to them; further researches will probably be made to clear up this point.

Those of our readers who are anti-tobacconists will read with satisfaction a paper just published by M. Decroix in the "*Bulletin de l'Association Francaise contre l'Abus du Tabac et de Boisons Alcooliques*," in which that gentleman enumerates no less than sixteen diseases—the list commencing with cancer of the tongue and ending with idiocy and premature old age—as resulting from the use of tobacco; and calculates that the loss to the State resulting from the absorption of capital in its cultivation, the time spent unproductively in smoking, and the money expended in the pursuit, capture, and maintenance of smugglers at 410 millions of francs, of which only 190 millions are recovered by taxation, leaving a balance of 220 millions of francs against the smokers, besides fires and a host of minor evils.—*London Times.*

